# Deletion of ABCB10 in beta-cells protects from high-fat diet induced insulin resistance

**DOI:** 10.1016/j.molmet.2021.101403

**Published:** 2021-11-23

**Authors:** Michael Shum, Mayuko Segawa, Raffi Gharakhanian, Ana Viñuela, Matthew Wortham, Siyouneh Baghdasarian, Dane M. Wolf, Samuel B. Sereda, Laura Nocito, Linsey Stiles, Zhiqiang Zhou, Vincent Gutierrez, Maike Sander, Orian S. Shirihai, Marc Liesa

**Affiliations:** 1 Department of Medicine, Division of Endocrinology, David Geffen School of Medicine at UCLA, 650 Charles E. Young Dr., Los Angeles, CA 90095, USA; 2 Department of Molecular and Medical Pharmacology, David Geffen School of Medicine at UCLA, 650 Charles E. Young Dr., Los Angeles, CA 90095, USA; 3 Molecular and Cellular Integrative Physiology, UCLA, 612 Charles E. Young Dr., Los Angeles, CA 90095, USA; 4 Bioscience Institute, Newcastle University, International Centre for Life, Central Parkway, Newcastle upon Tyne NE1 3BZ, United Kingdom; 5 Departments of Pediatrics and Cellular & Molecular Medicine, Pediatric Diabetes Research Center, University of California, San Diego, La Jolla, CA 92093, USA; 6 Evans Biomedical Research Center, Boston University School of Medicine, 650 Albany St., Boston, MA, 02118, USA; 7 Molecular Biology Institute at UCLA, 611 Charles E. Young Dr., Los Angeles, CA 90095, USA; 8 Department of Molecular Medicine, Faculty of Medicine, Universite Laval, Quebec City G1V 0A6, Canada

**Keywords:** Mitochondria, Insulin resistance, Beta-cell, ABCB10

## Abstract

**Objective:**

The contribution of beta-cell dysfunction to type 2 diabetes (T2D) is not restricted to insulinopenia in the late stages of the disease. Elevated fasting insulinemia in normoglycemic humans is a major factor predicting the onset of insulin resistance and T2D, demonstrating an early alteration of beta-cell function in T2D. Moreover, an early and chronic increase in fasting insulinemia contributes to insulin resistance in high-fat diet (HFD)-fed mice. However, whether there are genetic factors that promote beta-cell-initiated insulin resistance remains undefined. Human variants of the mitochondrial transporter *ABCB10*, which regulates redox by increasing bilirubin synthesis, have been associated with an elevated risk of T2D. The effects of T2D *ABCB10* variants on ABCB10 expression and the actions of ABCB10 in beta-cells are unknown.

**Methods:**

The expression of beta-cell *ABCB10* was analyzed in published transcriptome datasets from human beta-cells carrying the T2D-risk *ABCB10* variant. Insulin sensitivity, beta-cell proliferation, and secretory function were measured in beta-cell-specific ABCB10 KO mice (*Ins1*^*Cre*^*-Abcb10*^*flox/flox*^). The short-term role of beta-cell ABCB10 activity on glucose-stimulated insulin secretion (GSIS) was determined in isolated islets.

**Results:**

Carrying the T2Drisk allele G of *ABCB10* rs348330 variant was associated with increased *ABCB10* expression in human beta-cells. Constitutive deletion of *Abcb10* in beta-cells protected mice from hyperinsulinemia and insulin resistance by limiting HFD-induced beta-cell expansion. An early limitation in GSIS and H_2_O_2_-mediated signaling caused by elevated ABCB10 activity can initiate an over-compensatory expansion of beta-cell mass in response to HFD. Accordingly, increasing ABCB10 expression was sufficient to limit GSIS capacity. In health, ABCB10 protein was decreased during islet maturation, with maturation restricting beta-cell proliferation and elevating GSIS. Finally, *ex-vivo* and short-term deletion of *ABCB10* in islets isolated from HFD-fed mice increased H_2_O_2_ and GSIS, which was reversed by bilirubin treatments.

**Conclusions:**

Beta-cell ABCB10 is required for HFD to induce insulin resistance in mice by amplifying beta-cell mass expansion to maladaptive levels that cause fasting hyperinsulinemia.

## Highlights

• ABCB10 deletion in mouse beta-cells protects from HFD-induced insulin resistance.
• ABCB10 deletion increases mouse beta-cell function and limits HFD-induced beta cell expansion.
• T2D ABCB10 variant rs348330 is associated with increased ABCB0 expression in beta-cells.
• ABCB10 gain-of-function decreases glucose stimulated insulin secretion.

## 1 Introduction

The inherited risk of developing type 2 diabetes (T2D) is mostly associated with variants of genes that regulate beta-cell mass and insulin secretion [[1], [2], [3]]. However, not all T2D gene variants have demonstrated to cause insulinopenia or impair beta-cell function. Furthermore, different studies demonstrate that insulinopenia is not the only beta-cell-driven process that contributes to T2D [4]. As a result, increased fasting insulin concentrations precede the development of hyperglycemia caused by insulin resistance, with this elevation in fasting insulin occurring at normoglycemic values [5,6]. Given that healthy mature islets cannot sense the range of fasting normoglycemic concentrations [7,8], increased insulinemia at fasting normoglycemia has been explained by: i) decreased hepatic insulin clearance [9], ii) increased beta-cell mass leading to more insulin secretion at non-stimulatory glucose concentrations [10], and/or iii) beta-cell hypersensitivity to glucose, namely, the acquisition of secretory responsiveness to normoglycemic fasting glucose concentrations [11].

In mice, high-fat diet (HFD) feeding has been widely demonstrated to increase beta-cell mass and beta-cell sensitivity to low glucose concentrations. Exposure to high concentrations of non-esterified fatty acids (NEFA) is sufficient to increase the sensitivity of beta-cells to low glucose (3 mM) [11], by upregulating mitochondrial proton leak [12]. Moreover, beta-cell specific deletion of different genes that converge in lowering fasting insulinemia is sufficient to protect against age- and HFD-induced obesity and insulin resistance [10,13,14]. In one instance, protection from age-related insulin resistance is achieved through a primary decrease in insulin synthesis [14], which also limits HFD-induced beta-cell mass expansion and decreases fasting insulinemia [10]. In another instance, deleting glutamate dehydrogenase in beta-cells reduces the amplification of glucose stimulated insulin secretion (GSIS) and thus protects against insulin resistance caused by HFD [13].

Collectively, these studies support that genetic factors that elevate fasting insulinemia by excessively expanding beta-cell mass and/or increasing beta-cell sensitivity to *low fasting* glucose concentrations may initiate insulin resistance. Supporting the relevance of these mouse studies to humans, insulin replacement therapies induce insulin resistance in people with Type-1 diabetes [15]. This latter finding is of high importance, as it demonstrates that high insulin levels can drive insulin resistance independently of HFD feeding, obesity, and hyperglycemia [15]. The role of beta-cells in promoting insulin resistance is consistent with the observation that systemic insulin resistance precedes insulinopenia, with the inherited risk of developing T2D mostly involving genes that regulate islet function and mass [[1], [2], [3]]. Furthermore, the genetic predisposition of some lean individuals to develop T2D could be explained by beta-cell-initiated insulin resistance.

*ABCB10* is a mitochondrial transporter that exports biliverdin outside the mitochondria to increase bilirubin synthesis [16]. ABCB10-driven bilirubin synthesis has been shown to reduce both hepatocyte respiration as well as H_2_O_2_-stimulated insulin signaling in hepatocytes [16]. Multiple GWAS studies have consistently shown that *ABCB10* variants are associated with T2D [1,17,18], with the major allele G in *ABCB10* intronic variant rs348330 being the allele that confers T2D risk (p-value 3.37e-21, OR of 0.957; https://t2d.hugeamp.org/). However, the effect of this *ABCB10* T2D risk allele on *ABCB10* expression in beta-cells, as well as the role of ABCB10 in beta-cell function, are unknown. In this study, we cover these two gaps in knowledge by: i) executing an eQTL analysis in human beta-cells centered on the *ABCB10* rs348330 variant to determine the effects of carrying the allele G on *ABCB10* expression and ii) generating a beta-cell specific ABCB10 KO mouse and overexpressing ABCB10 *in vitro* to determine the role of ABCB10 in beta-cell mass expansion and function in response to HFD.

## 2 Materials and methods

### 2.1 Mice and diets

All experiments were approved by Institutional Animal Care and Use Committee at Boston University and by the Animal Research Committee at University of California, Los Angeles (UCLA). Constitutive beta-cell ABCB10 KO mice (βKO) were generated by breeding *Abcb10*^*flox/flox*^ mice in C57BL/6J background [16] with C57BL/6J-*Ins1*^*tm1.1(cre)Thor*^ procured from Jackson (stock # 026801) [19]. Breeding pairs were *Abcb10*^*WT/flox*;Ins1Cre(+/−)^ and *Abcb10*^*flox*/*flox*;^ ^Ins1Cre(−/−)^, from which WT (*Abcb10*^*flox/flox*;Ins1Cre(−/−)^; *Abcb10*^*wt/flox*;Ins1Cre(−/−)^), βHET (*Abcb10*^*flox/wt*;Ins1Cre(+/−)^), and βKO (*Abcb10*^*flox/flox*;Ins1Cre(+/−)^) littermates were analyzed. Despite the fact that βKO and βHET cohorts had the same WT littermates as controls, the βKO and βHET cohorts are presented in separated figures with the same WT controls because the phenotypes of βKO and βHET are different. Thorens et al. reported that *Ins1*^*Cre(+/−)*^mice showed normal glucose homeostasis and beta cell function *in vivo* [19], so cre-only controls were not analyzed in this study. Furthermore, Cre over-expression by adenoviral transduction had no effect on GSIS *ex vivo* (see methods 2.5), confirming the *in vivo* data from Thorens et al. [19]. Nonetheless, our studies do not rule out the possibility of a small effect of the Cre knock-in allele in our *in vivo* experiments. Taconic *created Abcb10 +/−* mice in a mixed C57BL/6J-129 background and we backcrossed them to C57BL/6J [20].

To induce obesity and insulin resistance, mice were fed a high-fat diet (HFD) with 45% kcal as fat *ad libitum* (#D12451, Research diets), starting after weaning (3–4 weeks of age). The length of HFD feeding was 14 weeks for the experiments consisting of *ex vivo Abcb10* deletion, 20 weeks for experiments with *Abcb10 +/−* mice, and 30 weeks in the case of βKO mice. For *ex vivo* deletion, the WT group consists of C57BL/6J mice that are not littermates and do not have any *Abcb10-*floxed alleles.

### 2.2 Glucose and insulin tolerance tests

In glucose tolerance tests (GTT), glucose in saline (1 g/kg) was injected intraperitoneally after an overnight fast (16 h). In insulin tolerance tests, insulin (Humulin 1/1000, 10 μl/g for males and 6.5 μl/g for females) was injected intraperitoneally after a 6 h fast. Using a FreeStyle Glucometer and strips, blood glucose was measured before (fasting glucose, time 0) and at different time points after injection.

### 2.3 Plasma proinsulin, insulin, and c-peptide

Blood was collected with capillary tubes coated with EDTA (Sarstedt) and the blood was centrifuged to collect plasma from overnight-fasted mice, as well as 15 and 30 min after glucose injection. Five to ten microliters of plasma were analyzed by ELISA to measure insulin and c-peptide (ALPCO), as well as proinsulin (Mercodia).

### 2.4 Islet isolation

The pancreata of CO_2_ euthanized mice were inflated through the bile duct with RPMI 11 mM glucose containing collagenase 1 mg/ml and then surgically removed. The collagenase-inflated pancreata were incubated for 10 min at 37 °C. RPMI with 11 mM glucose, 5% FBS, or 1% BSA was then used to inactivate and wash the collagenase. Histopaque and centrifugation separated the islets from the rest of the digested pancreas. Islets were manually picked and cultured in RPMI with 11 mM glucose, 10% FBS, and Penicillin/Streptomycin (P/S), as published [12].

### 2.5 Re-aggregated islet preparation and ex-vivo credelivery via adenoviral transduction

Twenty-four hours after isolation, islets were picked and digested with Accutase to obtain single cells. Cells were counted using a Neubauer chamber, with 2500–5000 cells plated per well of a 96 V-bottom-well plate (Nunc). Purified adenovirus (Vector Biolabs) encoding for Cre-RFP or for RFP alone (transduction control) were added to islet media (RPMI with 11 mM glucose, 10% FBS, P/S) at a MOI 200 for 2 h. The adenoviruses were washed 2 times with fresh islet media and the transduced single cells were cultured for 72 h in islet media to form re-aggregated islets. Efficiency of transduction was validated by live-cell fluorescence imaging (80–90% RFP-positive cells), and Cre-mediated excision was validated through the quantification of *Abcb1*0 mRNA levels by qPCR with TaqMan probes as published [20].

### 2.6 Insulin secretion *ex vivo*

Re-aggregated islets plated in a 96-well V bottom plate were incubated for 30 min at 37 °C in modified DMEM with 0.05% BSA (XF assay media, Agilent Technologies) with 2.8–3.8 mM glucose to measure basal secretion and with 15–16.7 mM glucose to measure GSIS. Islets were also co-incubated with 15–16.7 mM glucose + 40 mM KCl to assess K_ATP_independent insulin secretion. One re-aggregated islet (2500, 3000, or 5000 cells) was plated per well with triplicates and quadruplicates per mouse and experimental condition. The medium was collected and diluted to measure the amount of insulin secreted: 1/2 for basal secretion, 1/10−1/40 for GSIS and for GSIS + KCl to measure the amount of insulin secreted. A FRET-based method (HTRF, CisBio) was used to quantify insulin as reported previously reported [12].

### 2.7 Fluorescence imaging of re-aggregated islets

An Olympus DSU spinning disk microscope from the Boston University Imaging Core was used to perform live re-aggregated islet imaging at 37 °C and 5% CO_2_ in RPMI phenol-red free media at 11 mM glucose with 10% FBS. The cytosolic H_2_O_2_ (roGFP2-Orp1) reporter was delivered to re-aggregated islets using adenoviral transduction at MOI 200, following the same protocol as in Section 2.6. Thus, the MOI used for re-aggregated islets transduced with Cre was a total of 400 (co-transduction of MOI 200 for Cre-RFP, MOI 200 for roGFP2-Orp1). The wide fluorescence setting was used to collect the fluorescence intensity of 3–4 re-aggregated islets per mouse and condition. The average fluorescence intensity of oxidized roGFP2 (405 nm excitation, 509 nm emission) and reduced roGFP2 (488 nm excitation, 509 nm emission) per re-aggregated islet was quantified using Image J.

### 2.8 Oxygen consumption measurements in re-aggregated islets

One re-aggregated islet generated with 5000 cells was seeded in 1 μL of Matrigel on a poly-d-lysine–coated XF96 plate, as reported previously [12]. The XF96 plate was incubated for 3.5 min at 37 °C to solidify the Matrigel. Then, 100 μL of assay media (XF Minimal DMEM, pH 7.4, with 3 mM glucose and 0.1% FBS) was added per well, and the plate was incubated for ∼1 h at 37 °C before measuring oxygen consumption. High glucose (16.7 mM) was injected first, followed by Oligomycin (4.5 μM final, 10x in port) to block mitochondrial ATP synthesis and then FCCP (1 μM final) plus a nutrient supplement to achieve maximal respiration (11.4 mM sodium pyruvate and 2.9 mM of glutamine final). The last injection comprised the complex III inhibitor Antimycin A and the complex I inhibitor Rotenone to block mitochondrial electron transfer (2.5 μM final) [12].

### 2.9 Pancreas histology, beta-cell mass, and proliferation measurements

Mice were perfused with ice-cold PBS to remove the blood from the pancreas. The pancreas was carefully dissected and weighted before being placing in 10% neutral buffered formalin at 4 °C overnight. The sections analyzed covered the entire pancreas, with embedding and immunostaining performed at the TPLC core at UCLA. For beta-cell mass, paraffin-embedded sections incubated with insulin antibodies were stained with DAB. Images of the whole section were obtained with the Leica Aperio microscope at the TPLC core, from which total pancreas and islet areas (DAB-stained area) were quantified. The software Aivia v9.1 was used to quantify the percentage of Ki67-positive beta-cells, the latter identified by immunofluorescence as chromogranin-A positive cells, imaged using the Leica Ariol microscope at the TPLC core. An average filter was applied for the correction of blue (DAPI) channel intensity, and the pixel classifier tool was used to classify each channel/fluorophore. The red channel (chromogranin A) was used to colocalize and mask the other channels. Only the blue (DAPI) and green nuclei (Ki67) inside the red channel (beta cells) were segmented and counted.

### 2.10 Variant-centered analysis of ABCB10 expression in human beta-cells

*The ABCB1*0 mRNA content was analyzed using the human beta-cell expression datasets published by Viñuela et al. [21], which contain total expression measurements of 13 exons of *ABCB10*. As in the study conducted by Viñuela et al. InsPIRE was used to extract the eQTL information for all the associations with the *ABCB10* rs348330 (G/A) variant, with G being the T2D risk allele (p-value 3.37e-21, OR of 0.957; https://t2d.hugeamp.org/).

### 2.11 ABCB10 protein content in islets measured by mass spectrometry

The raw data for ABCB10 protein content were obtained from the dataset previously published by Wortham et al. [22]. Briefly, a total of 16 ABCB10 peptides were detected in total extracts from the islets of juvenile (4-week-old) and adult mice (1-year-old). To quantify ABCB10 peptides in juvenile and adult mice (unlabeled ^14^N peptides), each sample was combined 1:1 with total lysates of islets isolated from mice fed with a chow diet containing ^15^N-labeled amino acids, which achieved nearly complete labeling of islet proteins. Then, the ratios of heavy (^15^N) and light (^14^N) peptides were quantified using mass spectrometry. Thus, the ^14^N/^15^N ABCB10 peptide ratio provides a standardized quantification of ABCB10.

### 2.12 Adenoviral transduction of INS1 cells and GSIS measurements

INS-1 (832/13) cells were grown in RPMI 1640 medium with 10% FBS, 10 mM HEPES buffer, 1 mM pyruvate, 50 μM β-mercaptoethanol, 50 U/ml penicillin, and 50 μg/ml streptomycin. INS1-cells were transduced at MOI 5 with adenovirus encoding *Abcb10* generated as described [16]. Prior to GSIS, INS1 cells were cultured for 2 h in RPMI 1640 with 2 mM glucose without FBS. Cells were washed and incubated for 30 min in DMEM with 2 mM glucose and 0.05% BSA at pH 7.4 (XF assay media, Agilent Technologies). This was followed by a 60-min incubation of different groups of cells with the same media containing either 2 mM or 10 mM glucose. The media was stored at −20 °C until insulin measurements were performed. Insulin was measured by the HTRF insulin assay (Cisbio bioassays).

### 2.13 Liver triglyceride and qPCR measurements of the insulin receptor (Insr) in muscle

Liver triglycerides were measured as previously published [16]. Gastrocnemius muscle was homogenized in Trizol to extract RNA, and then retrotranscribed to cDNA. Sybr Green was used to quantify *Insrb* expression *using primers F: CCTGAAAAGTCACCTCCGTTCT and R: TTCAAGTATGCCATGCCATCA.*

## 3 Results

### 3.1 Beta-cell-specific deletion of ABCB10 protects from HFD-induced insulin resistance

ABCB10 exports biliverdin from the mitochondrial matrix to increase bilirubin synthesis in the cytosol, with bilirubin being a bioactive molecule that modulates redox signaling in hepatocytes [16]. However, the role of ABCB10 in beta-cells has not been determined to date. To this end, we generated beta-cell specific KO mice for *Abcb10* (βKO) by breeding *Abcb10*^*flox/flox*^ with *Ins1*^*Cre*^ mice [19]. We validated the efficiency of *Ins1*^*Cre*^-mediated deletion by measuring *Abcb1*0 mRNA content in islets isolated from *Abcb10*^*WT/flox;Ins1Cre+/-*^mice (βHET), as well as from βKO mice (Figure S1A). When fed a regular chow-diet, βKO male mice did not show differences in body weight (Figure S1B), fasting glycemia (Figure S1C), glucose tolerance (Figure S1D), and insulin sensitivity (Figure S1E). Therefore, ABCB10 expression in beta-cells is dispensable for glucose homeostasis in unstressed mice, similarly to what we reported for liver ABCB10 [16].

**Figure 1:**
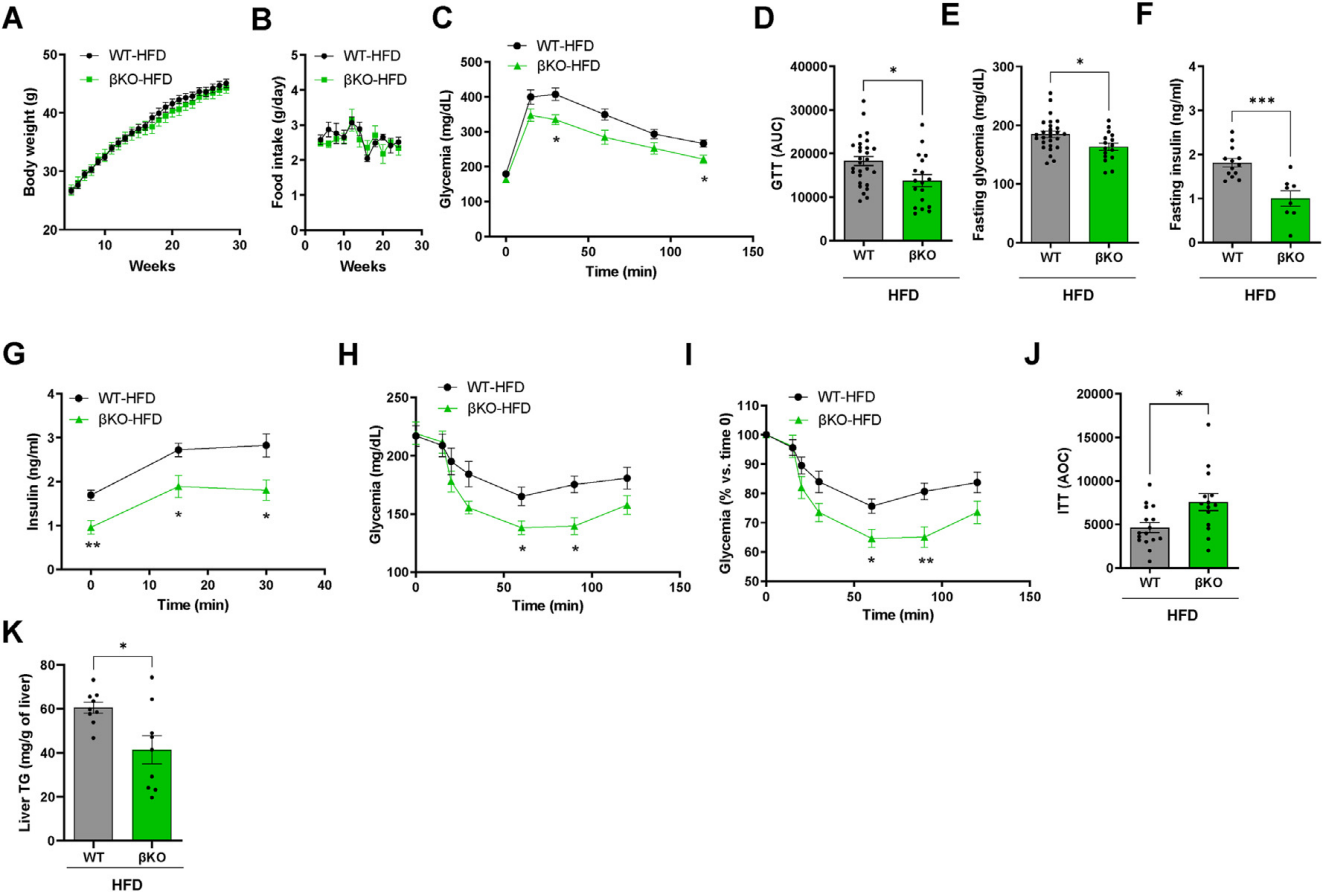
**ABCB10 deletion in beta-cells protects mice against HFD-induced insulin resistance without decreasing body weight**. (A–J) WT and βKO male mice fed a HFD for 30 weeks, n = 8–29 mice per group with error bars showing standard error of the mean (SEM). (A) Body weight, (B) Daily food intake measured every two weeks, (C) Blood glucose concentrations during glucose tolerance tests (GTT), ∗*P* < 0.05 Two-way ANOVA, Holm-Sidak's. (D) Area under the curve from the GTTs, ∗*P* < 0.05 Student's t-test, (E) Fasting glycemia,∗*P* < 0.05, Student's *t* test, (F) Fasting insulinemia, ∗∗∗*P* < 0.001 Student's t test, (G) Plasma insulin concentrations during GTTs, ∗*P* < 0.05; ∗∗*P* < 0.01, Two-way ANOVA, Holm-Sidak's. (H-J) Insulin tolerance tests measuring blood glucose after the intraperitoneal injection of insulin in mice fasted for 6 h (time 0) showed as (H) absolute glucose concentrations,∗*P* < 0.05,Two-way ANOVA, Holm-Sidak's, as (I) percent change over fasting glucose (time 0), ∗∗*P* < 0.01. Two-way ANOVA, Holm-Sidak's, and as (J) area over the curve (AOC), ∗*P* < 0.05, Student's *t* test. (K) Total triglyceride content in livers from WT and βKO male mice fed with a HFD for 30 weeks, n = 9 mice/group, ∗*P* < 0.05, Student's *t* test.

In marked contrast, after 30 weeks of high-fat diet (HFD) feeding, βKO mice showed an improvement in glucose tolerance, with lower fasting insulin and glucose concentrations, in the absence of changes in body weight and food intake (Figure 1A–F). In HFD-fed females, beta-cell ABCB10 deletion induced milder improvements in glucose homeostasis (Figure S2A–E). The milder improvement in females can be explained by HFD feeding not being as efficacious in inducing glucose intolerance and insulin resistance in females.

Given that protection from HFD-induced glucose intolerance in βKO mice occurred without changes in obesity and stemmed from a genetic change restricted to beta-cells, we expected that higher plasma insulin concentrations would explain the decrease in hyperglycemia. Contrary to our expectations, insulin concentrations were lower in βKO male mice at all time points measured during the GTTs (Figure 1G). Consequently, improved glucose tolerance in HFD-fed βKO male mice cannot be explained by elevated plasma insulin. Accordingly, insulin tolerance tests revealed that insulin sensitivity was improved in HFD-fed βKO male mice (Figure 1H–J), as well as in βKO females, but to a lesser extent (Figure S2F,G). Our findings are aligned with other studies demonstrating that beta-cell intrinsic changes resulting in increased fasting insulinemia contribute to insulin resistance induced by HFD [10,13,14]. The novelty of our mouse model is that it disassociates high-fasting insulin-driven insulin resistance from HFD-induced weight gain [10,13,14].

The absence of a decrease in body weight in HFD-fed βKO mice favors the fact that their improved insulin sensitivity stems from increased glucose disposal, rather than from an elevation in energy expenditure. Accordingly, transgenic expression of insulin in mice decreased glucose disposal without inducing weight gain [23]. Moreover, a recent preprint demonstrates that hyperinsulinemia decreases insulin receptor (*Insr*) transcription in muscle [24]. Thus, it was a possibility that higher *Insr* expression in the muscle of HFD-fed βKO mice could explain their improved glucose disposal. However, we found that *Insr* mRNA content was not increased in the muscles of HFD-fed βKO mice and even showed a trend to be decreased when compared to chow-diet fed mice (Figure S3A). As a result, the benefits in insulin sensitivity induced by beta-cell ABCB10 deletion could not be explained by higher *Insr* expression in muscle. We then measured triglyceride accumulation in the liver, which is a hallmark of hyperinsulinemia, as well as of systemic insulin resistance. We found that triglyceride content in the livers of HFD-fed βKO mice was significantly decreased when compared to that in WT mice (Figure 1K). Consequently, these latter data support the hypothesis that improved liver metabolism may explain the improvement in glycemia and systemic insulin sensitivity induced by beta-cell ABCB10 deletion.

### 3.2 Beta-cell-specific ABCB10 deletion limits beta-cell expansion induced by HFD, without decreasing basal beta-cell proliferation or glucose-stimulated insulin secretion (GSIS)

**Figure 2:**
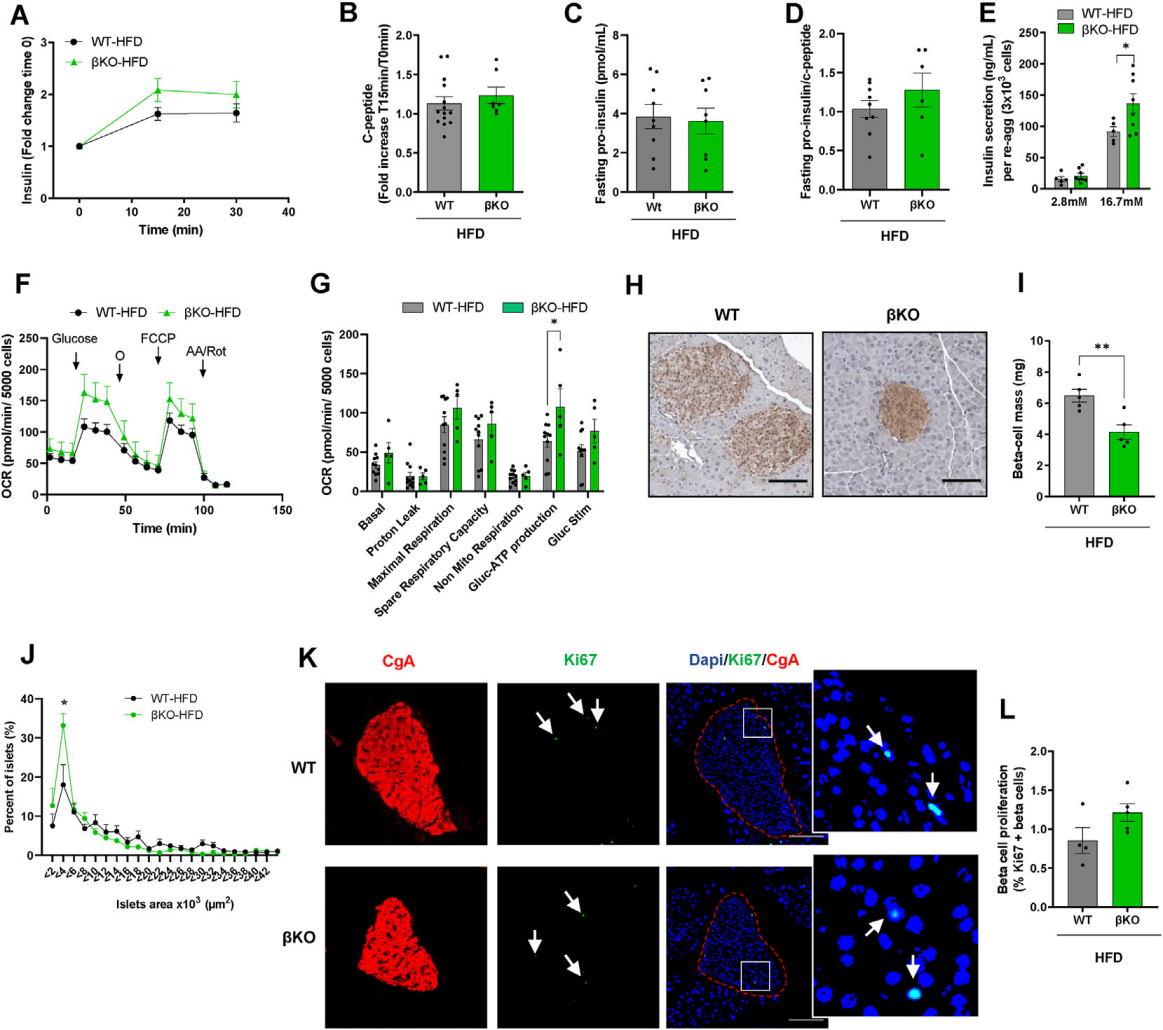
**Decreased fasting insulinemia induced by ABCB10 deletion is explained by a limitation in beta-cell mass expansion, but not by a decrease in basal beta-cell proliferation or GSIS capacity per beta-cell**. (A–H) WT and βKO male mice were fed a HFD diet for 30 weeks and error bars represent standard error of the mean. (A) Fold change in plasma insulin, n = 10–16 mice/group and (B) c-peptide during the GTTs in HFD-fed WT and βKO male mice, n = 7–14 mice/group. (C) Fasting proinsulin and (D) proinsulin/c-peptide ratio in plasma from HFD-fed WT and βKO male mice, n = 6–9 mice/group. (E) Glucose-stimulated insulin secretion (GSIS) measured *ex vivo* in re-aggregated islets, with re-aggregation normalizing the number of cells per islet and genotype, n = 5–8 mice/group, ∗*P* < 0.05, Student's *t* test. (F) Mitochondrial respirometry trace showing oxygen consumption (pmols O2/min) of 1 re-aggregated islet per well (5000 cells, each mouse in triplicate, error bars are SEM) under 3 mM glucose (Basal), in response to 16.7 mM glucose (Glucose), to the mitochondrial ATP synthase inhibitor oligomycin (o), to a mitochondrial uncoupler (FCCP) used to assess maximal respiratory capacity, and to antimycin A/rotenone (AA/ROT) used to block mitochondrial respiration. (G) Bar graphs averaging independent respirometry experiments with n = 5–11 mice per group, with ATP-synthesizing respiration being calculated by subtracting oligomycin-insensitive respiration from glucose-stimulated respiration.∗*P* < 0.05, Student's *t* test. (H) Representative image of a paraffin-embedded section covering the entire pancreas, with beta-cells stained using an anti-insulin antibody and DAB, scale bar 100 μm. (I) Quantification of beta-cell mass by determining beta-cell area, n = 5 mice/group, ∗*P* < 0.05, Student's *t* test. (J) Islet size distribution curves obtained from panels H and I, plotting the percentage of islets in the Y axis binned according to area values listed in the X axis, n = 5 mice/group, ∗*P* < 0.05∗∗*P* < 0.01 and ∗∗∗*P* < 0.001 Two-way ANOVA, Holm-Sidak's. (K) Representative immunofluorescence images of sections covering the entire pancreas and stained with Ki-67 (green, replication), DAPI (nucleus), and chromogranin A (red, beta-cell label) to measure beta-cell replication in response to HFD, scale bar 100 μm. (L) Average of % Ki67+/chromogranin A+ cells in each mouse, n = 4–5 mice/group.

Beta-cell-restricted genetic manipulations that limit HFD-induced beta-cell mass expansion or the amplification of glucose-stimulated insulin secretion (GSIS) have been shown to protect against insulin resistance [10,13,14]. Thus, we investigated whether changes in GSIS capacity and beta-cell mass in βKO male mice could explain their protection from HFD-induced insulin resistance. In an *in vivo* measurement of GSIS, we quantified the fold-increase in insulin over fasting concentrations during the GTTs. HFD-fed βKO mice showed a trend to have a higher fold-increase in insulin after glucose injections (Figure 2A). However, as hepatic insulin clearance is reduced by HFD feeding, and clearance is a major determinant of plasma insulin concentrations, c-peptide measurements are needed to confirm changes in beta-cell secretory function *in* *vivo* [25]. The same c-peptide response was seen in HFD-fed βKO mice after glucose injection (Figure 2B), which supported that the GSIS improvement *in vivo* could be mild.

An elevation of proinsulin concentration in plasma, which is not cleared by the liver, is a sign of beta-cell dysfunction. Further supporting that ABCB10 deletion did not induce beta-cell dysfunction in HFD-fed mice, fasting proinsulin content (Figure 2C) and the fasting proinsulin/c-peptide ratio (Figure 2D, Figure S3B) were not changed in HFD-fed βKO male mice.

The absence of changes in the proinsulin:c-peptide ratio supports that changes in beta-cell mass, rather than changes in beta-cell secretory function, drive the decrease in circulating insulin content in βKO male mice. To further confirm this conclusion, we isolated islets from HFD-fed WT and βKO male mice and dispersed them into single cells, as we published [12]. This approach allowed us to generate cell-number matched WT and βKO re-aggregated islets and thus assess whether changes in GSIS capacity existed that were independent of the potential changes in islet cell numbers. We observed close to a 40% increase in GSIS capacity in re-aggregated islets from HFD-fed βKO mice (Figure 2E), which further demonstrated that a decrease in GSIS capacity per beta-cell cannot explain lower insulin concentrations *in vivo* (Figure 1G). In addition, these data support the hypothesis that βKO beta-cells are healthier as their functional capacity is elevated.

Mitochondrial ATP synthesis by oxidative phosphorylation is a major determinant of GSIS. Thus, to understand the mechanism underlying elevated GSIS, we measured the effects of ABCB10 deletion on mitochondrial respiratory function. A significant increase was observed in ATP-synthesizing respiration after glucose stimulation (Figure 2F,G), indicating that the increase in GSIS in βKO re-aggregated islets could be explained by increased ATP synthesis. Furthermore, both maximal respiratory capacity and respiration under high glucose showed a trend to improve in βKO re-aggregated islets (Figure 2F,G). These trends are aligned with literature showing that the contribution of decreased beta-cell mitochondrial respiration to T2D can go beyond a reduction in the acute production of ATP needed for GSIS [26]. Thus, the respirometry profile in βKO re-aggregated islets further supports that beta-cells are healthier in the absence of ABCB10, or less T2D-like.

We then determined whether a limitation in beta-cell mass expansion induced by HFD-feeding could explain lower circulating insulin content in βKO mice *in vivo*. Similar to what was reported in mice with limited insulin synthesis [10], HFD-fed βKO male mice showed a reduction in total beta-cell mass (Figure 2H,I), as measured by immunohistochemistry in paraffin-embedded sections covering the entire pancreas. It is important to emphasize that HFD feeding can expand beta-cell mass by three different processes: i) increasing basal beta-cell proliferation, as shown by a higher percentage of proliferating beta-cells per islet [10]; ii) causing transient bursts of beta-cell proliferation that precede [27] and follow insulin resistance induced by HFD (1 and 10 weeks of HFD) [28]; and iii) causing an increase in individual beta-cell size (12 weeks of HFD) [28].

Consequently, we determined whether decreased islet size and basal beta-cell replication (Ki67+) could explain the limitation in beta-cell expansion induced by *Abcb10* deletion. Islet size distribution curves, plotting the percentage of islets in the Y axis binned according to islet area values listed in the X axis, were markedly shifted to the left in HFD-fed βKO mice (Figure 2J). This left shift demonstrated that ABCB10 deletion limited islet hyperplasia, as it preserved a higher number of smaller islets when compared to HFD-fed WT islets (Figure 2H,I). However, HFD-fed βKO islets showed a similar percentage of proliferating beta-cells (Ki67+/chromogranin A+ cells) (Figure 2K,L). Altogether, these data support the hypothesis that *Abcb10* deletion prevents HFD-induced beta-cell expansion without affecting basal beta-cell replication.

### 3.3 High ABCB10 expression in beta-cells is associated with lower GSIS capacity in mice and with a higher risk of developing T2D in humans

**Figure 3:**
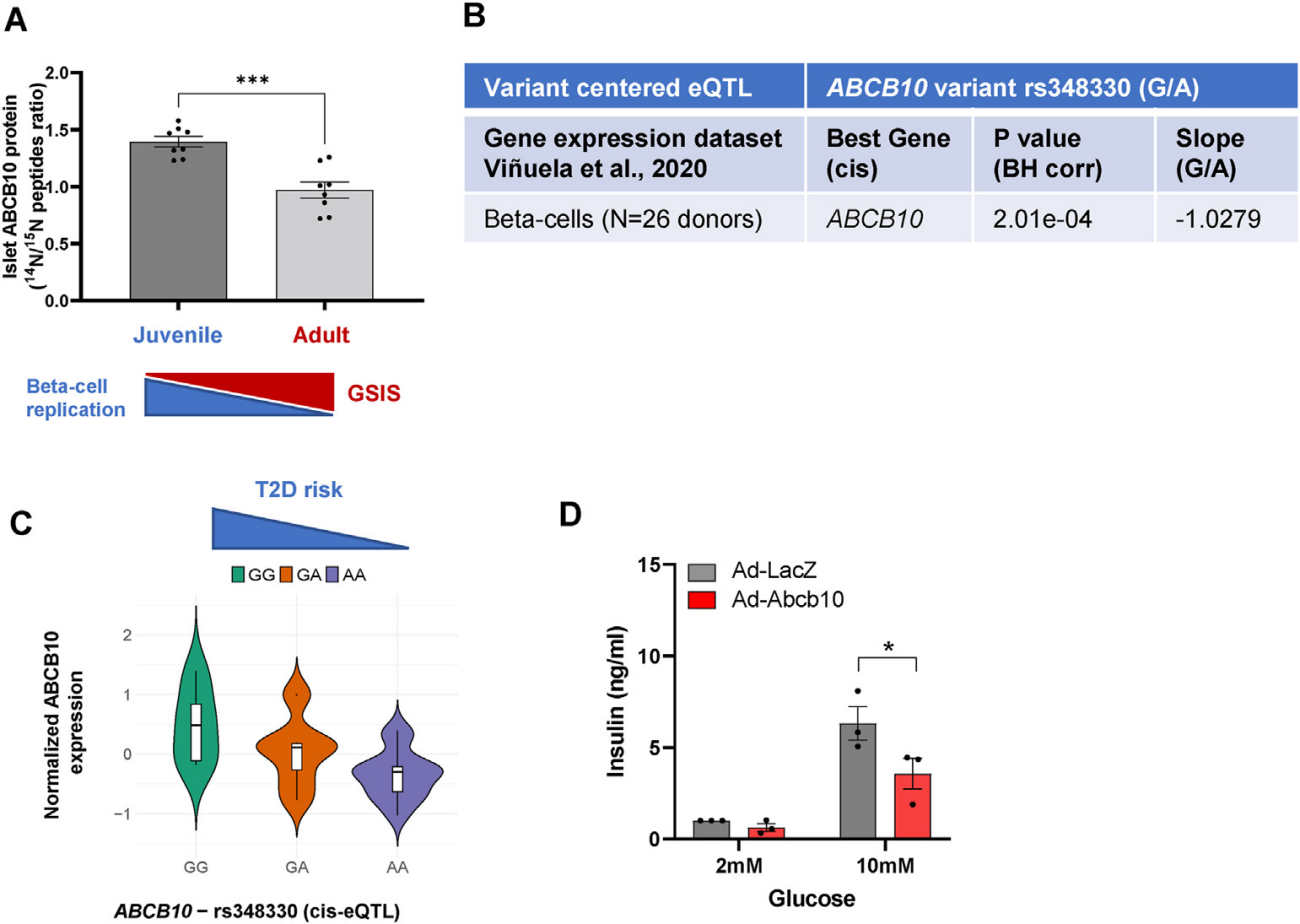
**ABCB10 expression is negatively associated with GSIS capacity in humans and mice**. (A) Mouse ABCB10 protein content measured by mass spectrometry in adult islets (1-year-old) characterized by high GSIS and low beta-cell replication, when compared to juvenile islets (4-weeks-old) with low GSIS and high beta-cell replication, *n* = 8 per group, ∗∗∗*P* < 0.001,Student's *t* test. (B) Variant-centered eQTL analyses from the human beta-cell gene expression dataset by Viñuela et al., 2020 [19] with n = 26 donors, finding that ABCB10 is the best gene associated with *ABCB10* variant rs348330 and that ABCB10 expression is significantly correlated with the allele carried in variant rs348330, with a slope of −1.02 (negative value meaning that G carriers show higher expression). P values are Benjamini & Hochberg (BH) adjusted. (C) Violin plot from the dataset in panel B showing ABCB10 expression levels in GG, GA, and AA carriers in T2D-risk *ABCB10* variant rs348330. (D) GSIS measured in INS1 cells with ABCB10 gain-of-function achieved via adenoviral transduction (LacZ, control adenovirus), n = 3 independent experiments, ∗*P* < 0.05, Two-way ANOVA, Holm-Sidak's.

We next aimed to determine whether beta-cell ABCB10 protein content is regulated during physiological processes that change beta-cell mass and GSIS capacity. A recent study compared the proteome of juvenile islets from 4-week-old mice to adult islets from 1-year-old mice [22]. Remarkably, during this 11-month period of islet development and maturation, GSIS capacity is doubled, while basal beta-cell proliferation is decreased [22]. The ABCB10 total protein content in juvenile islets was 44% higher (p = 0.002) than in adult islets (Figure 3A), demonstrating that ABCB10 protein content is decreased during islet maturation. Thus, under physiological conditions, ABCB10 protein content is positively associated with replication but negatively associated with GSIS capacity.

In the pathological context of T2D in humans, *ABCB10* variant rs348330 (G/A) has been consistently associated with a higher risk to develop T2D (p-value 3.37e-21, OR of 0.957), with this significant association to T2D being preserved after adjusting for obesity (BMI) (p-value 2.709e-8, OR of 0.9484) [1,17,18]. However, rs348330 is an intronic variant, which means that the different nucleotides constituting the rs348330 variant cannot directly change ABCB10 amino-acid composition. Therefore, we determined whether carrying the rs348330 T2D risk variant (G allele) could be associated with changes in *ABCB1*0 mRNA content.

To this end, we performed expression Quantitative Trait Loci (eQTL) analyses, which is the most stringent test to determine the gene whose expression is most affected by a noncoding GWAS variant. To perform these eQTL analyses on rs348330, we used published exon-sequencing transcriptome datasets of isolated beta-cells from 26 human donors [21]. A total of 13 *ABCB10* exons were detected and quantified, which were used to determine *ABCB1*0 mRNA content (Supplementary Table 1). Our eQTL analyses revealed that *ABCB10* was the best associated gene with the GWAS variant rs348330 (G/A), with the correlation of the content of the best (in terms of p value) associated *ABCB10* exon with rs348330 showing a negative slope value of −1.09 (Figure 3B). The negative value shows that the carriers of the major allele (G) express higher levels of ABCB10 in beta-cells. Accordingly, this slope was calculated by comparing the content of the best *ABCB10* associated exon to rs348330 in subjects carrying the two copies of the major allele (GG) in rs348330, versus subjects carrying GA, and versus AA carriers (Figure 3C).

Further confirming that r348330 G/G is associated with elevated ABCB10 expression, the content of all 13 ABCB10 exons showed negative slope values that ranged between −1.02 and −0.40. Moreover, the association of the content of 12 out of 13 *ABCB10* exons with r348330 was statistically significant (p < 0.05, Benjamini& Hochberg adjusted) (Supplementary Table 1). Given that the G allele confers a higher T2D risk [18] and that GG carriers showed higher ABCB10 expression in beta-cells (Figure 3B,C), these data support the hypothesis that increased ABCB10 function in beta-cells could increase the risk of developing T2D.

As the proteomics and transcriptomics data suggested that elevated ABCB10 function could be suppressing GSIS, we determined GSIS capacity in INS1-cells transduced with LacZ (control) or *Abcb10*-encoding adenovirus. We found that ABCB10 overexpression induced a 40% decrease in GSIS (Figure 3D). Altogether, these results support that elevated ABCB10 function is sufficient to decrease GSIS.

### 3.4 Halving beta-cell ABCB10 expression improves glucose tolerance in HFD-fed mice by increasing beta-cell GSIS capacity without changing insulin sensitivity

**Figure 4:**
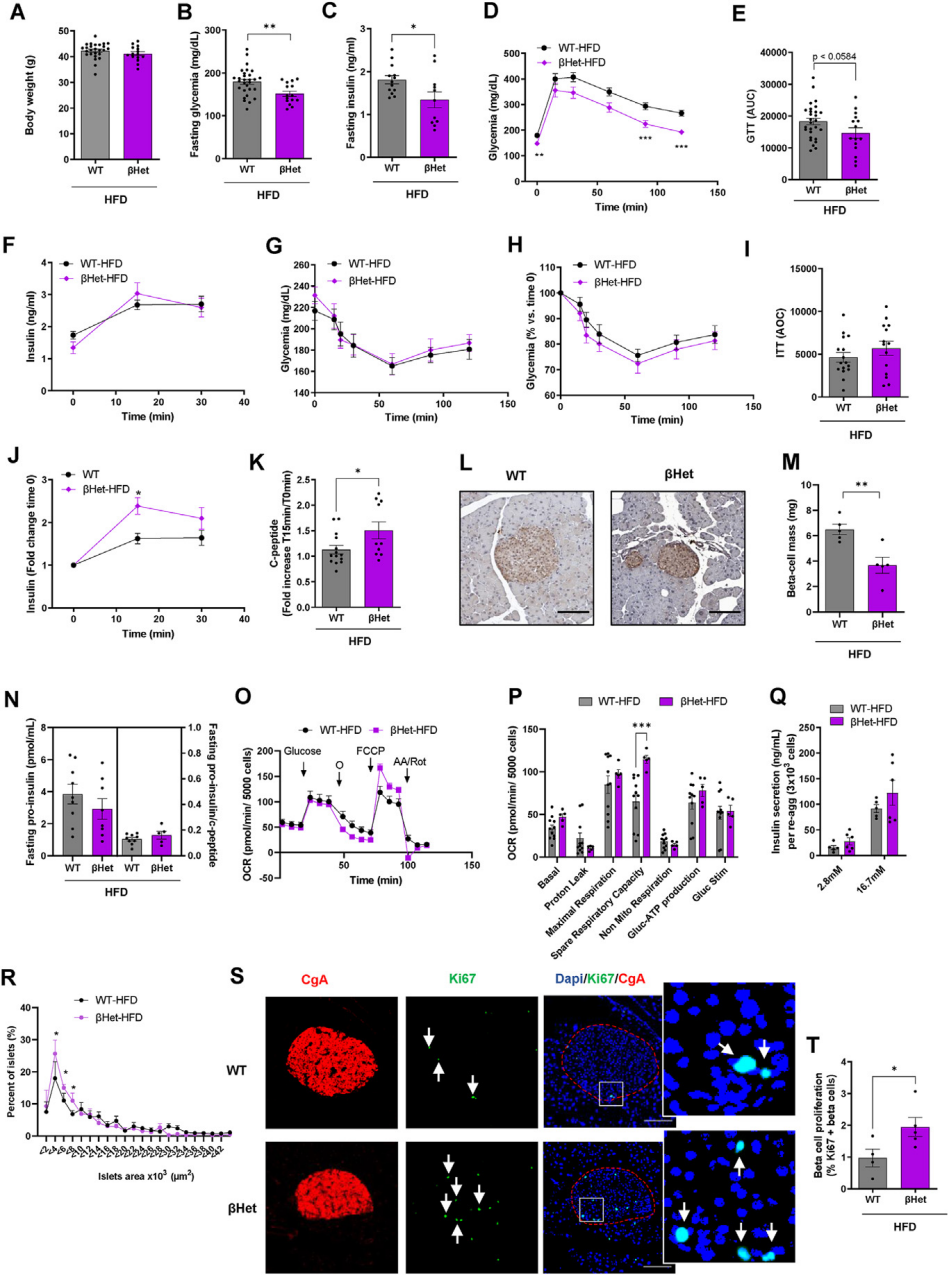
**Halving beta-cell ABCB10 expression improves glucose tolerance by increasing beta-cell GSIS capacity *in vivo*, without changing insulin sensitivity**. (A-K)WT and βHET male mice were fed a HFD for 30 weeks, n = 10–29 mice/group, error bars represent SEM. (A) Body weight, (B) Fasting blood glycemia, and (C) Fasting plasma insulinemia at time 0 of the glucose tolerance test (GTT), ∗*P* < 0.05, ∗∗*P* < 0.01, Student's *t* test. (D–E) Blood glucose measurements during the GTT in response to the intraperitoneal injection of glucose (1 g/kg) after o/n fasting, plotted as (D) Absolute glucose concentrations, ∗∗*P* < 0.01 and ∗∗∗*P* < 0.001, Two-way ANOVA, Holm-Sidak's and (E) Area under the curve, p = 0.0584, Student's *t* test. (F) Plasma insulin concentrations in response to glucose injections (1 g/kg) measured during the GTTs. (G–I) Insulin tolerance tests in mice fasted for 6 h and plotting (G) Absolute blood glucose, (H) % blood glucose over fasting concentrations, and (I) Area over the curve. (J) Fold change in plasma insulin and in (K) c-peptide concentrations in response to glucose injections (1 g/kg) measured during the GTTs. ∗*p* < 0.05, Two-way ANOVA, Holm-Sidak's. (L) Representative image of a paraffin-embedded section covering the entire pancreas from WT and βHET male mice fed with a HFD for 30 weeks, with beta cells stained using DAB and an anti-insulin antibody. Scale bar 100 μm. (M) Beta-cell mass was quantified by determining total beta-cell area (insulin stain) in sections covering the whole pancreas, n = 5 mice/group, ∗∗*P* < 0.01, Student's *t* test, error bars represent SEM. (N) Fasting proinsulin and proinsulin/c-peptide ratio in plasma from HFD-fed WT and βHET male mice, n = 5–9 mice per group, SEM. (O) Mitochondrial respirometry trace showing oxygen consumption (pmols O_2_/min) of re-aggregated islets (5000 cells per well, each mouse in triplicate, error bars are SEM) under 3 mM glucose (Basal), in response to the injection of 16.7 mM glucose (Glucose), the inhibitor of mitochondrial ATP synthesis oligomycin (o), an uncoupler (FCCP) to assess maximal respiratory capacity, and antimycin A/rotenone (AA/ROT), which block mitochondrial electron transfer. (P) Bar graphs averaging independent respirometry experiments, with ATP-synthesizing respiration being calculated by subtracting oligomycin insensitive respiration from glucose-stimulated respiration, *n* = 5–11 mice/group, ∗∗∗*P* < 0.001, Student's *t* test, error bars represent SEM. (Q) Glucose-stimulated insulin secretion (GSIS) measured in re-aggregated islets after 30 min of incubation with 15–16.7 mM glucose, with re-aggregation equalizing the number of cells per islet and genotype, *n* = 5–6 mice per group. Error bars represent SEM. (R) Islet size distribution measured from the sections used to determine beta-cell mass, n = 5 mice/group,∗*P* < 0.05, Two-Way ANOVA, Holm-Sidak's, error bars represent SEM. (S) Representative immunofluorescence analysis of pancreata sections stained with Ki67, co-localized with chromogranin A to measure beta-cell replication in response to HFD. Scale bar 100 μm. (T) Quantification of the % Ki67+/chromogranin A+ cells in each mouse (n = 4–5 mouse/group), ∗*P* < 0.05, Student's *t* test, error bars represent SEM.

We then determined whether decreasing beta-cell ABCB10 expression in a more physiological range (50%) induced changes in beta-cell function *in vivo*. To this end, we analyzed glucose homeostasis and beta-cell function in βHET mice littermates from the same HFD-fed WT and βKO cohorts analyzed (see breeding strategy in methods). HFD-fed βHET mice showed improved glucose tolerance when compared to WT mice, as well as a decrease in fasting glycemia and insulinemia (Figure 4A–E). The improvement in glucose tolerance induced by halving beta-cell ABCB10 expression was not explained by protection from obesity, as βHET mice had no changes in body weight (Figure 4A).

Consequently, halving ABCB10 expression in beta-cells induced similar benefits in glycemia when compared to beta-cell ABCB10 deletion (Figure S4A–D). However, the measurements of insulin during the GTTs revealed a highly remarkable qualitative difference between βHET and βKO mice. Plasma insulin concentrations of βHET males after glucose injections showed a trend to be higher than that in WT male mice (Figure 4F), which meant that insulin concentrations in βHET males after glucose injection almost doubled those of βKO (Figure S4E). Moreover, insulin tolerance tests demonstrated that βHET males were not protected from insulin resistance (Figure 4G–I), as the βKO were (Figure S4F,G). Consistent with preserved insulin resistance, βHET males were not as significantly protected from hepatic steatosis as βKO mice were (Figure S4H). Importantly, no significant differences in the fat pad weights were observed between WT, βHET, and βKO mice (Figure S4I). These results further support the hypothesis that resistance to fat gain was not the mechanism underlying improved glucose tolerance in both βHET and βKO mice.

Thus, the expectation was that elevated insulin output in response to high glucose would be responsible for the improvement in glucose tolerance in βHET mice. Confirming this conclusion, the fold increase in both plasma insulin and c-peptide concentrations in response to the injections of glucose was significantly higher in βHET mice, when compared to WT mice (Figure 4J,K). Remarkably, mice with one allele of *Abcb10* deleted in all tissues (*Abcb10* +/− mice) presented a similar response to HFD-feeding. *Abcb10* +/− mice showed improved glucose tolerance with higher GSIS *in vivo* (Figure S5A–D). This latter data was reveals that the improvement in beta-cell function in *Abcb10 +/-* mice can contribute to combating HFD-induced hyperglycemia even when the *Abcb10* genetic dosage is halved in all tissues.

To elucidate the beta-cell process driving the increase in circulating insulin and c-peptide content in HFD-fed βHET mice, we measured both beta-cell mass and GSIS capacity in re-aggregated islets *ex-vivo*. We found a 43% reduction in beta-cell mass in HFD-fed βHET mice (Figure 4L,M), which was consistent with decreased fasting insulin concentrations. Fasting proinsulin concentrations showed a trend to be decreased in βHET male mice without changing the proinsulin/c-peptide ratio (Figure 4N). This latter data confirms that the decrease in beta-cell mass observed in βHET is not a sign of beta-cell dysfunction. We then determined the capacity of βHET re-aggregated islets to respire and execute GSIS *ex vivo*. We found that re-aggregated islets from βHET mice had a significant increase in spare respiratory capacity, a sign of better mitochondrial health and resilience (Figure 4O,P). In addition, βHET re-aggregated islets showed the same rates of glucose stimulated ATP-synthesizing respiration (Figure 4O,P), which was consistent with the unexpected absence of a significant increase in GSIS measured *ex vivo* (Figure 4Q). This GSIS *ex vivo* data is in marked contrast with the significant increase in GSIS detected *in vivo* in βHET mice (Figure 4Q vs. Figure 4J,K).

An important difference in the measurements of GSIS *in vivo* versus *ex vivo* is that the dispersion−re-aggregation procedure eliminates the differences in total beta-cell counts that can occur *in vivo*. Therefore, an increase in the number of smaller and functional beta-cells in βHET mice could reconcile decreased fasting insulin and increased GSIS *in vivo*, with lower beta-cell mass and unchanged GSIS capacity per beta cell *ex vivo*. In agreement with this conclusion, we found that the number of smaller islets was increased in βHET mice (Figure 4R), together with a doubling of the percentage of Ki67 positive cells (Figure 4S,T) when compared to WT mice. Consequently, the decrease in beta-cell mass in βHET islets was not explained by a decrease in beta-cell proliferation but rather by a decrease in islet size.

### 3.5 Ex vivo and short-term deletion of ABCB10 in islets enhances redox-stimulated GSIS, which is reversed by bilirubin treatment

Beta-cells are highly adaptive, with their size, proliferation, and capacity to secrete insulin changing in response to age, diet, and circulating glucose concentrations. Therefore, some of the changes in beta-cell function and mass observed in HFD-fed βHET and βKO mice will be a result of their improved glycemia. In other words, all the beta-cell phenotypes observed are not exclusively explained by the absence of beta-cell autonomous ABCB10 actions but by the long-term consequences of preventing severe hyperglycemia.

The fact that no phenotypes are observed in chow-diet-fed βKO mice demonstrates that ABCB10 is neither required nor deleterious for beta-cell development and function in lean C57BL/6J mice. Thus, one can conclude that ABCB10 only becomes maladaptive in beta-cells when ABCB10 activity is excessively increased in response to HFD, similar to what was published in hepatocytes [16]. This excessive elevation in ABCB10 activity can cause an early decrease in insulin output, which can signal the recruitment of an excessive expansion of beta cell mass to compensate for this hindered insulin output. As a result, we aimed to determine whether the short-term and cell-autonomous changes induced by ABCB10 deletion could regulate beta-cell function selectively in HFD-fed mice, independent of HFD-induced beta-cell mass expansion. The expectation was that such an approach would determine whether changes in beta-cell function could initiate the phenotype observed in HFD-fed βHET and βKO mice.

**Figure 5:**
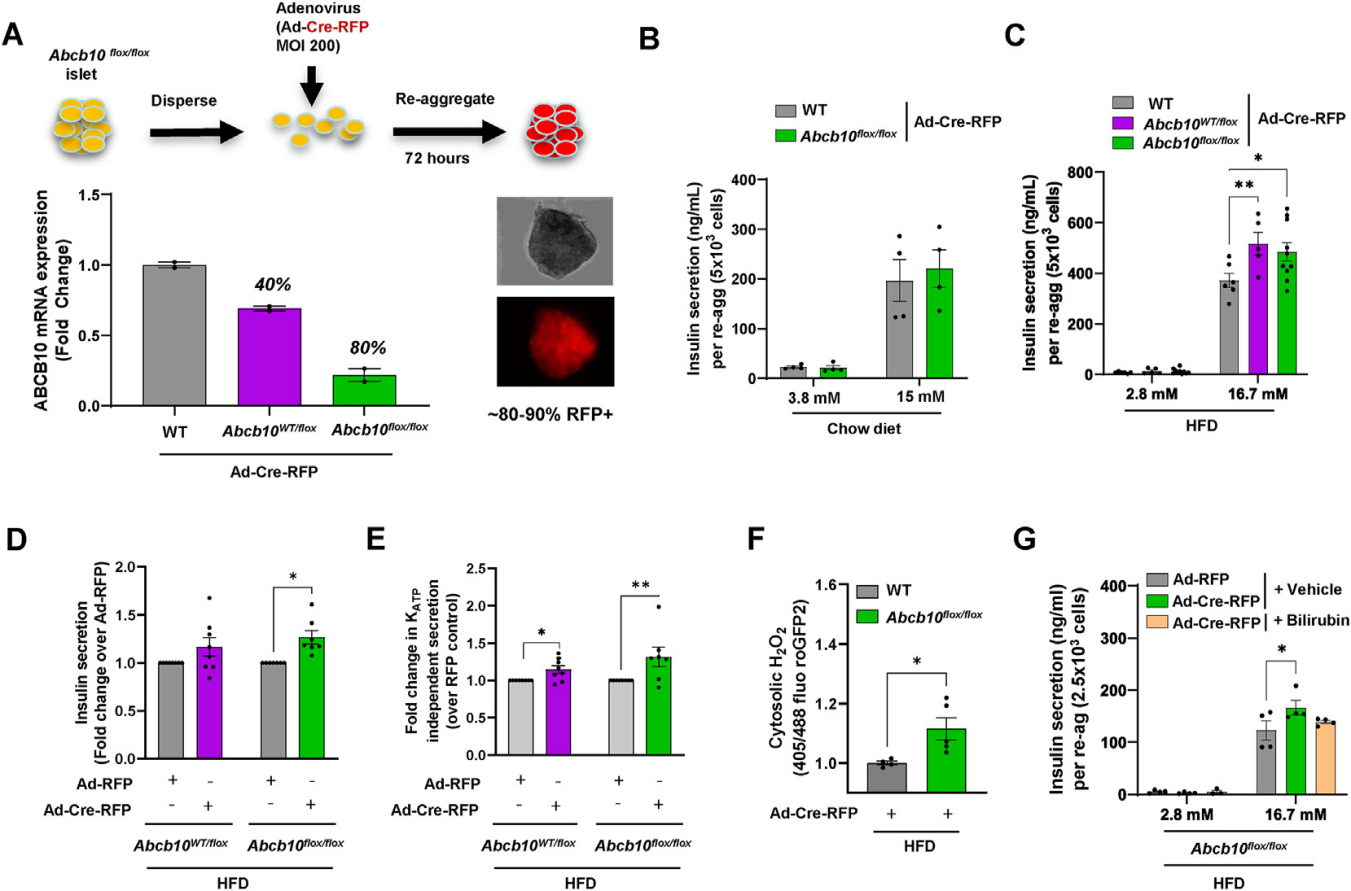
**Short-term deletion of ABCB10 in re-aggregated islets enhances redox-stimulated GSIS, which is reversed by bilirubin treatment**. (A–G) Re-aggregated islets with *Abcb10-*floxed alleles were deleted *ex vivo* via adenoviral transduction of dispersed WT (*Abcb10*^*WT/WT*^), *Abcb10*^*WT/flox*^, and *Abcb10*^*flox/flox*^ islets with adenovirus encoding Cre-RFP or control adenovirus just encoding RFP. (A) Scheme with dispersion−reaggregation transduction (MOI = 200) procedure, with a representative bright field and wide fluorescence image of a re-aggregated islets transduced with Cre-RFP, together with qPCR measurements of *Abcb10* expression. n = 2 mice per group, error bars represent SD. (B) Effects of *ex vivo Abcb10* deletion on GSIS in re-aggregated islets isolated from chow-diet-fed mice (14 weeks), n = 4 mice/group in 2 independent islet isolations with error bars representing SEM. (C–D) Effects of halving and deleting *Abcb10* expression on GSIS of re-aggregated islets from HFD-fed mice, using as a control (C) WT (*Abcb10*^*WT/WT*^) re-aggregated islets transduced with Cre-RFP, ∗p < 0.05, ∗∗p < 0.01, Student's *t* test vs. WT Cre or (D) *Abcb10*^*flox/WT*^and *Abcb10*^*flox/flox*^re-aggregated islets transduced with RFP. n = 5–10 mice/group in 4 independent islet isolations. Error bars represent SEM. ∗p < 0.05 Student's *t* test vs. RFP. (E) Effects of halving and deleting *Abcb10* on K+_ATP_ independent insulin secretion (under 40 mM KCl and 16.7 mM glucose) in re-aggregated islets from HFD-mice, when compared to their paired controls, namely half of the dispersed cells transduced with RFP. n = 7–9 mice per group in 4 independent islet isolations. Error bars represent SEM. ∗p < 0.05 Student's *t* test vs. RFP. (F) Live-cell imaging of cytosolic H_2_O_2_ using roGFP2-Orp1 sensors in re-aggregated islets from HFD-mice under stimulatory concentrations of glucose (11 mM) with *ex vivo* deletion of *Abcb10* compared to WT (*Abcb10*^*WT/WT*^)-Cre-RFP controls. n = 4–5 mice per group. ∗p < 0.05, Student's *t* test. Error bars represent SEM. (G) The effects of treatment with bilirubin 10 μM overnight on GSIS in re-aggregated islets from HFD-fed mice and with *Abcb10* deleted *ex vivo*, n = 4 mice/group, ∗p < 0.05, Two-Way ANOVA, Holm Sidak's, error bars represent SEM.

We developed an approach to delete *Abcb10 ex-vivo*, which allowed us to eliminate *Abcb10* after the occurrence of HFD-induced beta-cell mass expansion, as well as to assess the cell-autonomous effects of *Abcb10* deletion on beta-cell function. To this end, we first isolated islets from WT, *Abcb10*^*flox/flox*^, and *Abcb10*^*wt/flox*^mice and dispersed them into single cells. Dispersion allows adenoviruses encoding for Cre recombinase and red fluorescent protein (RFP) to reach cells located at the core of the islet, resulting in a marked improvement in adenoviral transduction efficiency (Figure 5A). An adenovirus encoding for RFP alone was used as a transduction control. After transduction, these single islet cells were allowed to re-aggregate for 72 h, which provides sufficient time for Cre to excise *Abcb10* and decrease *Abcb10* expression. This approach effectively induced an 80% decrease in *Abcb10* expression, which was consistent with 80–90% transduction efficiency as observed by RFP + cells (Figure 5A). Similar to what we observed *in vivo*, *ex vivo* deletion of *Abcb10* in re-aggregated islets from chow-diet-fed mice did not induce any changes in GSIS (Figure 5B).

We then used the same approach to examine *Abcb10 ex-vivo* in isolated islets from mice fed with HFD for 14 weeks. The rationale for choosing 14 weeks is that HFD-induced proliferative bursts and hyperplasia of beta-cells have already occurred after 12 weeks of feeding [28]. In addition, mice fed with a HFD for 14 weeks still show elevated GSIS capacity *in vivo*, meaning that 14 weeks of feeding is still in the relatively early adaptive phase of beta-cells to HFD feeding [28]. Accordingly, we reproduced, *ex vivo*, the increase in GSIS capacity induced by HFD reported *in vivo* (Figure 5B versus Figure 5C).

*Ex vivo* deletion of one or two *Abcb10* alleles was sufficient to induce a 26% increase in GSIS, when compared to islets from HFD-fed WT mice transduced with Cre-RFP (Figure 5C). Of note, we observed a better correlation between the magnitude of the increase in GSIS and the number of *Abcb10* alleles deleted when we used a paired RFP control (Figure 5D). Briefly, the paired control consists of half of the islet cells from the same mouse, but transduced with RFP instead of Cre-RFP (Figure 5D). Moreover, GSIS was still increased when re-aggregated islets with *Abcb10* deleted or halved were exposed to high glucose in combination with 40 mM KCl (Figure 5E). This latter result supports the hypothesis that short-term deletion of ABCB10 does not elevate insulin secretion by enhancing ATP synthesis under high glucose. Rather, ABCB10 deletion improves GSIS in a K_ATP_ channel closure independent manner. This conclusion is indicated by our data showing that KCl exposure and its concomitant depolarization of the plasma membrane does not equalize secretion between WT and KO. In other words, secretion should be equalized by KCl exposure if the differences stemmed from ATP-mediated K_ATP_ channel closure.

Our previous study in hepatocytes revealed that *Abcb10* deletion decreased bilirubin synthesis, resulting in improved H_2_O_2_-mediated insulin signaling [16]. The reason is that bilirubin can directly scavenge H_2_O_2_ and even generate a H_2_O_2_ scavenging redox cycle with biliverdin [29]. H_2_O_2_ released from mitochondria at physiological concentrations in islets was shown to be a signal amplifying the K_ATP_-independent component of GSIS [30]. Thus, we aimed to determine the H_2_O_2_-scavenging role of bilirubin in the short-term actions of ABCB10 on GSIS amplification. By co-transducing Cre-encoding adenovirus together with adenovirus encoding for a cytosolic H_2_O_2_ reporter (roGFP2-Orp1), we first determined the effects of short-term *Abcb10* deletion on cytosolic H_2_O_2_ in re-aggregated islets isolated from HFD-fed mice. We detected an increase in cytosolic H_2_O_2_ of *Abcb10* KO re-aggregated islets under stimulatory glucose (Figure 5F). Then, we treated *Abcb10* KO re-aggregated islets with bilirubin 10 μM and found that bilirubin treatment reversed the increase in GSIS induced by short-term *Abcb10* deletion (Figure 5G). This result indicates that a decrease in bilirubin actions caused by *Abcb10* deletion was responsible for the increase in GSIS.

## 4 Discussion

Variants of genes that regulate beta-cell mass and function are the main source of inherited susceptibility to T2D [[1], [2], [3]]. Therefore, the capacity of beta-cells to adapt and counteract hyperglycemia induced by insulin resistance was deemed to be the key inherited trait determining T2D development. However, recent preclinical studies demonstrate that beta-cell restricted genetic manipulations that primarily decrease fasting insulinemia protect mice against insulin resistance [13,14]. Thus, these are proof-of-concept studies supporting the existence of an earlier and broader role of beta-cells in T2D development [10,13,14]. It is then possible that some of the untested T2D-risk variants of genes that control beta-cell mass are contributing to T2D development by inducing insulin resistance.

We propose that genetic variants that facilitate an excessive upregulation of beta-cell ABCB10 expression will confer a higher risk of developing T2D. In agreement with this proposal, we find that the T2D risk allele (G) in *ABCB10* variant rs348330 is associated with higher ABCB10 expression in human beta-cells. From these data, we speculate that individuals carrying the (G) allele in rs348330 will show an excessive elevation in beta-cell ABCB10 expression in response to high caloric diets, resulting in an exaggerated beta-cell mass expansion that elevates fasting insulinemia and concomitant insulin resistance. This speculation stems from our mouse data showing that beta-cell-specific ABCB10 deletion in mice limits HFD-induced beta-cell mass expansion, while protecting against fasting hyperinsulinemia and insulin resistance.

In addition to potentially causing fasting hyperinsulinemia to initiate insulin resistance, increased beta-cell ABCB10 expression in humans could elevate T2D risk by limiting GSIS capacity as well. Supporting this conclusion, βHET mice had a lower fasting hyperinsulinemia and improved glucose tolerance, but they were not protected against insulin resistance like βKO mice. Rather, the improvement in glucose tolerance of βHET mice was explained by higher GSIS and beta-cell function *in vivo*. Thus, one could conclude that decreased beta-cell mass and fasting hyperinsulinemia in βHET mice just reflected their improved glycemic control, stemming from a better adaptability of their beta-cells to insulin resistance. As a result, it is likely that humans having lower beta-cell ABCB10 expression and a lower T2D risk are better modeled by βHET mice. The reason is that complete deletion of ABCB10 does not occur in humans carrying the T2D-risk intronic variant rs348330.

In agreement with the role of ABCB10 in regulating beta-cell proliferation and adaptation in physiology, ABCB10 protein content is effectively downregulated during islet maturation in mice, with mature islets acquiring high GSIS capacity and losing their ability to proliferate. Therefore, one can conclude that the physiological role of beta-cell ABCB10 expression is to ensure a favorable redox state that facilitates beta-cell replication early in life. Then, to acquire maximal GSIS capacity, islet maturation requires a downregulation of ABCB10 activity. In terms of ABCB10-mediated redox control, we previously published that ABCB10 exports biliverdin out of the mitochondria to increase cytosolic bilirubin synthesis [16], with bilirubin synthesis consuming cytosolic NADPH and bilirubin itself directly scavenging H_2_O_2_. In this regard, the high abundancy of bilirubin (1−10 μM) in the blood can explain why ABCB10-driven bilirubin synthesis is dispensable for islet development and function in control mice. Beta-cells can still have bilirubin available from the bloodstream, as well as bilirubin synthesized from biliverdin generated in the beta-cell cytosol. Another option is that other redox players could compensate for the need for bilirubin synthesis driven by ABCB10 activity in certain microdomains (mitochondria-ER connections).

A major process shown to explain higher GSIS in adult islets, when compared to juvenile islets, is elevated cytosolic NADPH production [22]. It is a possibility that ABCB10 protein content is downregulated during maturation to prevent ABCB10-driven bilirubin synthesis from consuming cytosolic NADPH, with this NADPH being available to maximize GSIS. In this context, we propose that excessive ABCB10 activity induced at the early stages of HFD feeding will mildly decrease the insulin output by limiting GSIS. The decrease in insulin output at this early stage of HFD feeding can still be counteracted by expanding beta-cell mass. However, in the presence of high ABCB10 activity, such expansion can be excessive and maladaptive. In agreement with this conclusion, our data shows that ABCB10 overexpression *in vitro* is sufficient to decrease GSIS without increasing secretion at low glucose. Moreover, while *ex vivo* deletion of ABCB10 is inert to islets from chow-diet-fed mice, *Abcb10* deletion is sufficient to increase GSIS in isolated islets from HFD-fed mice. In other words, *Abcb10* deletion can still increase GSIS after beta-cell mass expansion has already occurred. This improvement in GSIS excludes that ABCB10 deletion was inducing beta-cell death in HFD-fed islets, as if this were the case, GSIS *ex vivo* should be decreased.

The fact that GSIS was still higher when KCl was added together with high glucose supports that *Abcb10* deletion could be augmenting GSIS by elevating cytosolic NADPH production. However, we observed that ABCB10 deletion increased cytosolic H_2_O_2_, which would not be expected with elevated NADPH availability. H_2_O_2_ was shown to be a signaling molecule that amplifies GSIS by a still uncharacterized molecular mechanism [30,31]. Moreover, bilirubin scavenges H_2_O_2_ and thus can decrease H_2_O_2_ independently of NADPH availability [29]. Our data showing that bilirubin supplementation reverses the increase in GSIS induced by ABCB10 deletion advocates that the actions on H_2_O_2_-mediated signaling, rather than on cytosolic NADPH content, are responsible for ABCB10-driven changes in GSIS. If higher NADPH availability was responsible for increased GSIS in ABCB10 KO re-aggregated islets, bilirubin supplementation should not decrease GSIS. The reason is that bilirubin supplementation would liberate the endogenous NADPH used to synthesize bilirubin from biliverdin and, as a result, should increase GSIS.

We propose that these ABCB10 actions controlling H_2_O_2_-mediated signaling are responsible for the differences in beta-cell replication between βHET and βKO mice. Complete deletion of ABCB10 will cause a larger decrease in bilirubin synthesis, which will increase the availability of H_2_O_2_ molecules destined for signaling, when compared to a 50% reduction of ABCB10. Mild increases in cytosolic H_2_O_2_ concentrations have been shown to stimulate beta-cell replication, while higher increases in the physiological range of H_2_O_2_ concentrations have been shown to stop beta-cell proliferation without causing cell death [32]. Therefore, it is a possibility that higher H_2_O_2_-mediated signaling in βKO, as expected by lower bilirubin synthesis when compared to βHET, explains the absence of increased beta-cell replication in βKO mice *in vivo*.

In all, our study demonstrates that ABCB10 in beta cells is a key determinant of the response of an organism to high caloric diets, playing a maladaptive role that promotes hyperglycemia by limiting GSIS and increasing fasting insulinemia.

## Author contributions

M. Shum and M. Segawa performed and designed experiments and collected and analyzed data. M.L. conceived the project, performed, and designed experiments, wrote the first draft of the manuscript, and acquired funds. A.V. performed the eQTL analyses of *ABCB10* expression. M.W. and M. Sander performed ABCB10 proteomic analyses in islets. S.B. and S.B.S assisted with islet isolations, islet respirometry, and *ex vivo* insulin secretion measurements. L.S., L.N, D.M.W, Z.Z, and V.G. assisted with mouse experiments. R.G. performed histology, beta-cell mass measurements, and mouse experiments. O.S.S provided resources and discussed data.
