# Supplementary material for: Deletion of ABCB10 in beta-cells protects from high-fat diet induced insulin resistance
Source: Mol Metab. 2021 Nov 23;55:101403. doi: 10.1016/j.molmet.2021.101403 (PMC8689243; doi:10.1016/j.molmet.2021.101403)
Supplement: Multimedia component 1 [file mmc1.pptx]

## Slide 1
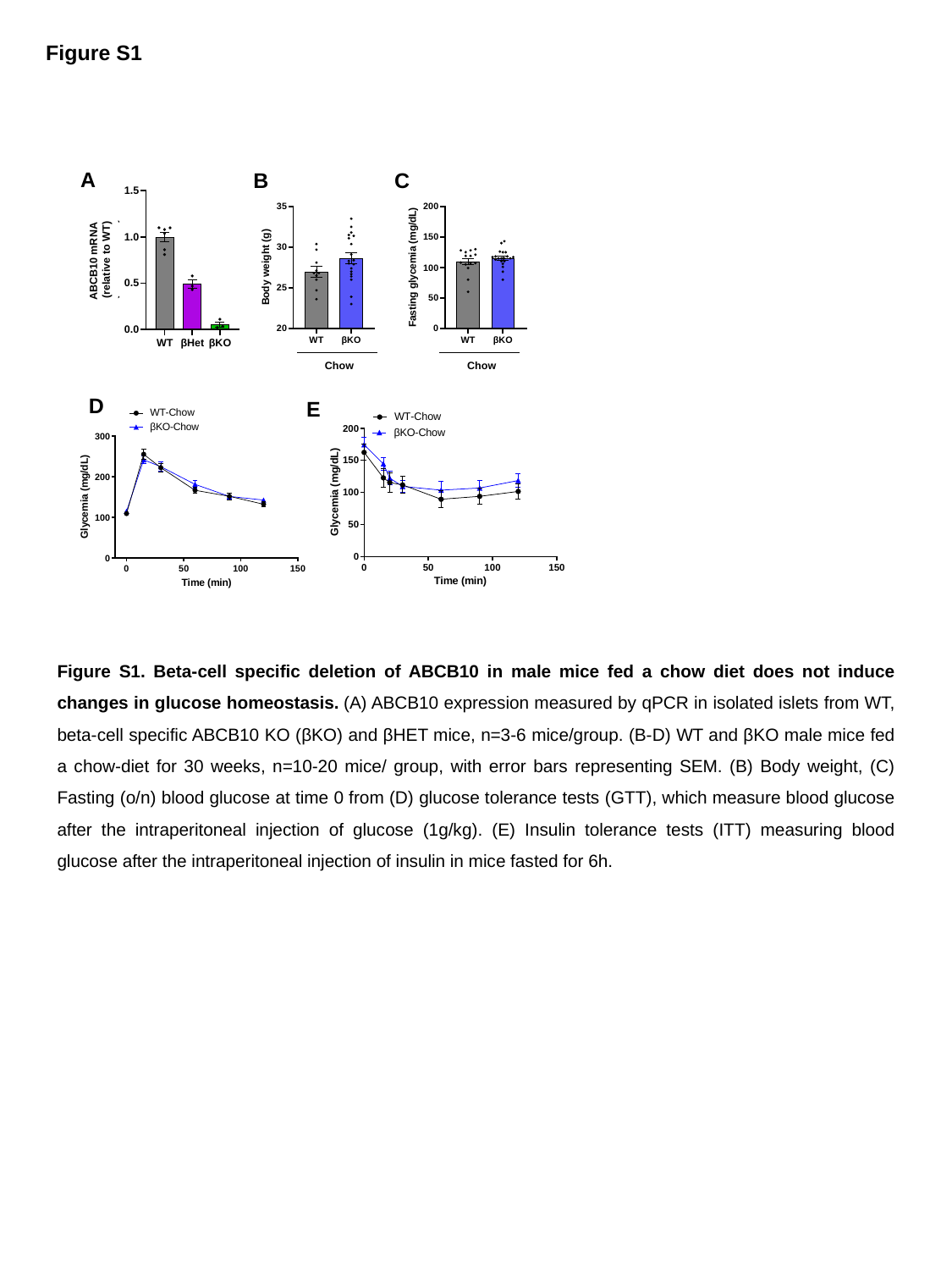

Figure S1
A
C
B
ABCB10 mRNA
(relative to WT)
Chow
Chow
D
E
Figure S1. Beta-cell specific deletion of ABCB10 in male mice fed a chow diet does not induce changes in glucose homeostasis. (A) ABCB10 expression measured by qPCR in isolated islets from WT, beta-cell specific ABCB10 KO (βKO) and βHET mice, n=3-6 mice/group. (B-D) WT and βKO male mice fed a chow-diet for 30 weeks, n=10-20 mice/ group, with error bars representing SEM. (B) Body weight, (C) Fasting (o/n) blood glucose at time 0 from (D) glucose tolerance tests (GTT), which measure blood glucose after the intraperitoneal injection of glucose (1g/kg). (E) Insulin tolerance tests (ITT) measuring blood glucose after the intraperitoneal injection of insulin in mice fasted for 6h.

## Slide 2
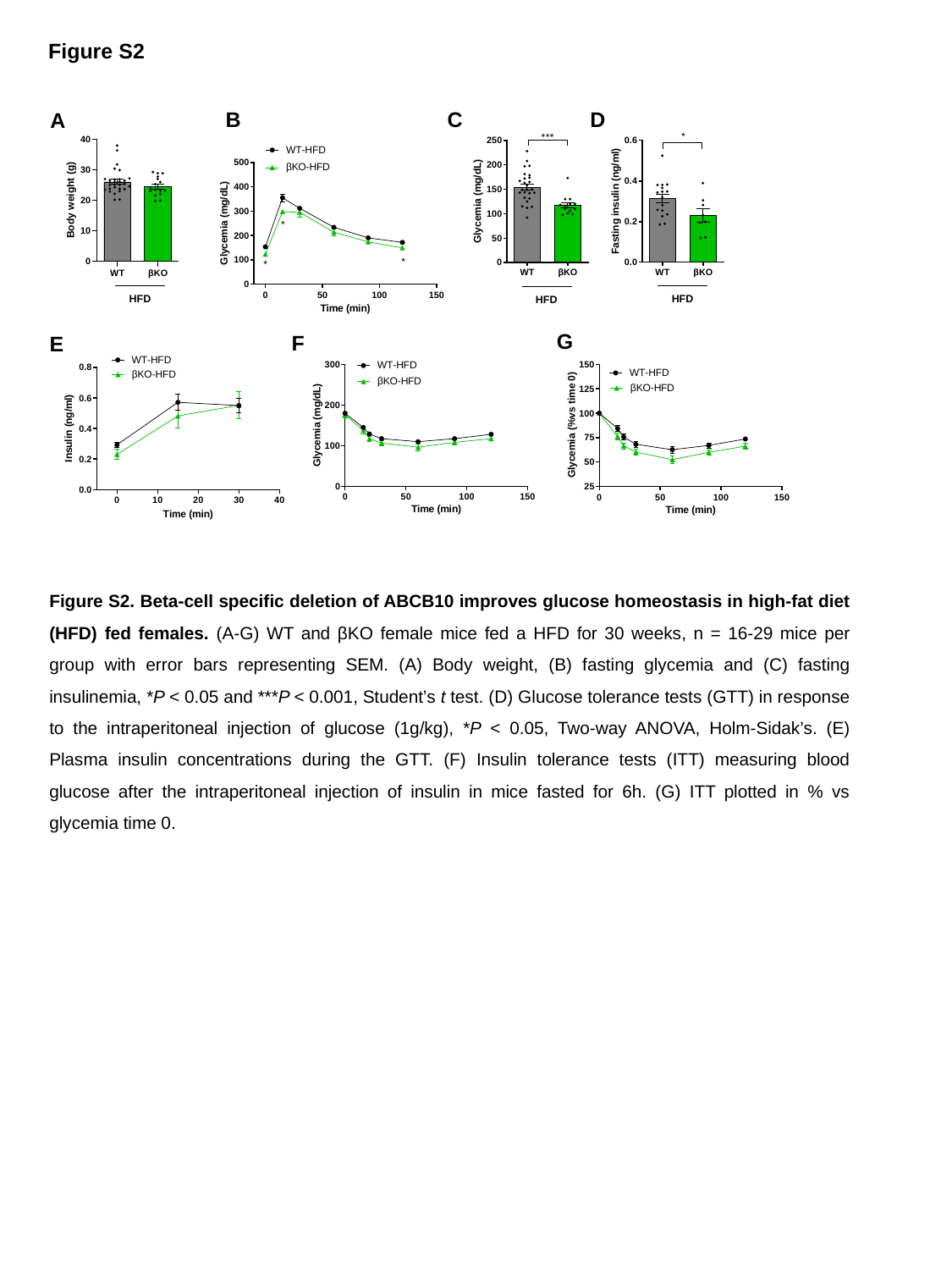

Figure S2
B
C
D
A
HFD
HFD
HFD
G
F
E
Figure S2. Beta-cell specific deletion of ABCB10 improves glucose homeostasis in high-fat diet (HFD) fed females. (A-G) WT and βKO female mice fed a HFD for 30 weeks, n = 16-29 mice per group with error bars representing SEM. (A) Body weight, (B) fasting glycemia and (C) fasting insulinemia, *P < 0.05 and ***P < 0.001, Student’s t test. (D) Glucose tolerance tests (GTT) in response to the intraperitoneal injection of glucose (1g/kg), *P < 0.05, Two-way ANOVA, Holm-Sidak’s. (E) Plasma insulin concentrations during the GTT. (F) Insulin tolerance tests (ITT) measuring blood glucose after the intraperitoneal injection of insulin in mice fasted for 6h. (G) ITT plotted in % vs glycemia time 0.

## Slide 3
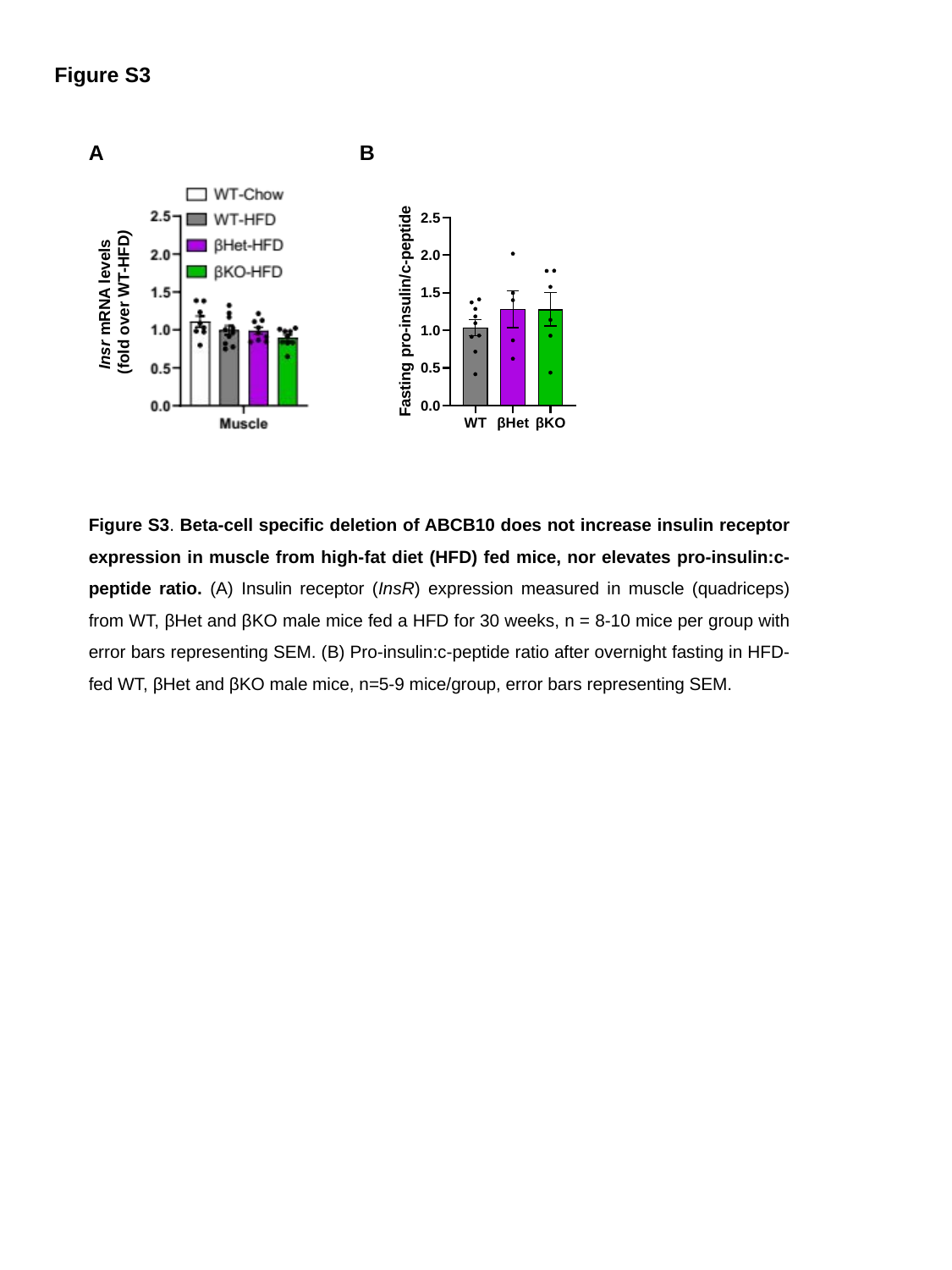

Figure S3
B
A
Insr mRNA levels
(fold over WT-HFD)
Figure S3. Beta-cell specific deletion of ABCB10 does not increase insulin receptor expression in muscle from high-fat diet (HFD) fed mice, nor elevates pro-insulin:c-peptide ratio. (A) Insulin receptor (InsR) expression measured in muscle (quadriceps) from WT, βHet and βKO male mice fed a HFD for 30 weeks, n = 8-10 mice per group with error bars representing SEM. (B) Pro-insulin:c-peptide ratio after overnight fasting in HFD-fed WT, βHet and βKO male mice, n=5-9 mice/group, error bars representing SEM.

## Slide 4
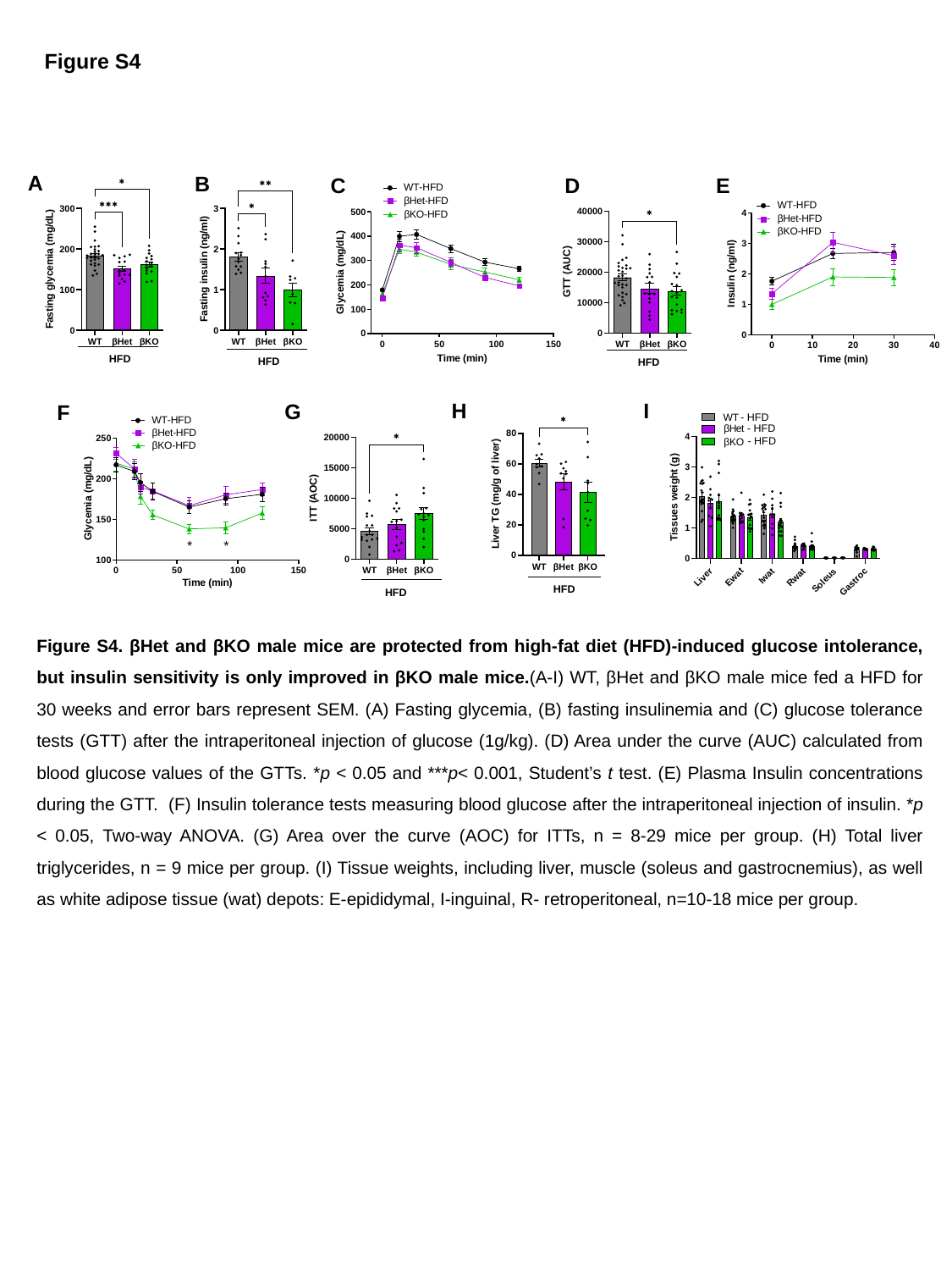

Figure S4
A
B
C
E
D
HFD
HFD
HFD
I
H
G
F
- HFD
- HFD
- HFD
HFD
HFD
Figure S4. βHet and βKO male mice are protected from high-fat diet (HFD)-induced glucose intolerance, but insulin sensitivity is only improved in βKO male mice.(A-I) WT, βHet and βKO male mice fed a HFD for 30 weeks and error bars represent SEM. (A) Fasting glycemia, (B) fasting insulinemia and (C) glucose tolerance tests (GTT) after the intraperitoneal injection of glucose (1g/kg). (D) Area under the curve (AUC) calculated from blood glucose values of the GTTs. *p < 0.05 and ***p< 0.001, Student’s t test. (E) Plasma Insulin concentrations during the GTT. (F) Insulin tolerance tests measuring blood glucose after the intraperitoneal injection of insulin. *p < 0.05, Two-way ANOVA. (G) Area over the curve (AOC) for ITTs, n = 8-29 mice per group. (H) Total liver triglycerides, n = 9 mice per group. (I) Tissue weights, including liver, muscle (soleus and gastrocnemius), as well as white adipose tissue (wat) depots: E-epididymal, I-inguinal, R- retroperitoneal, n=10-18 mice per group.

## Slide 5
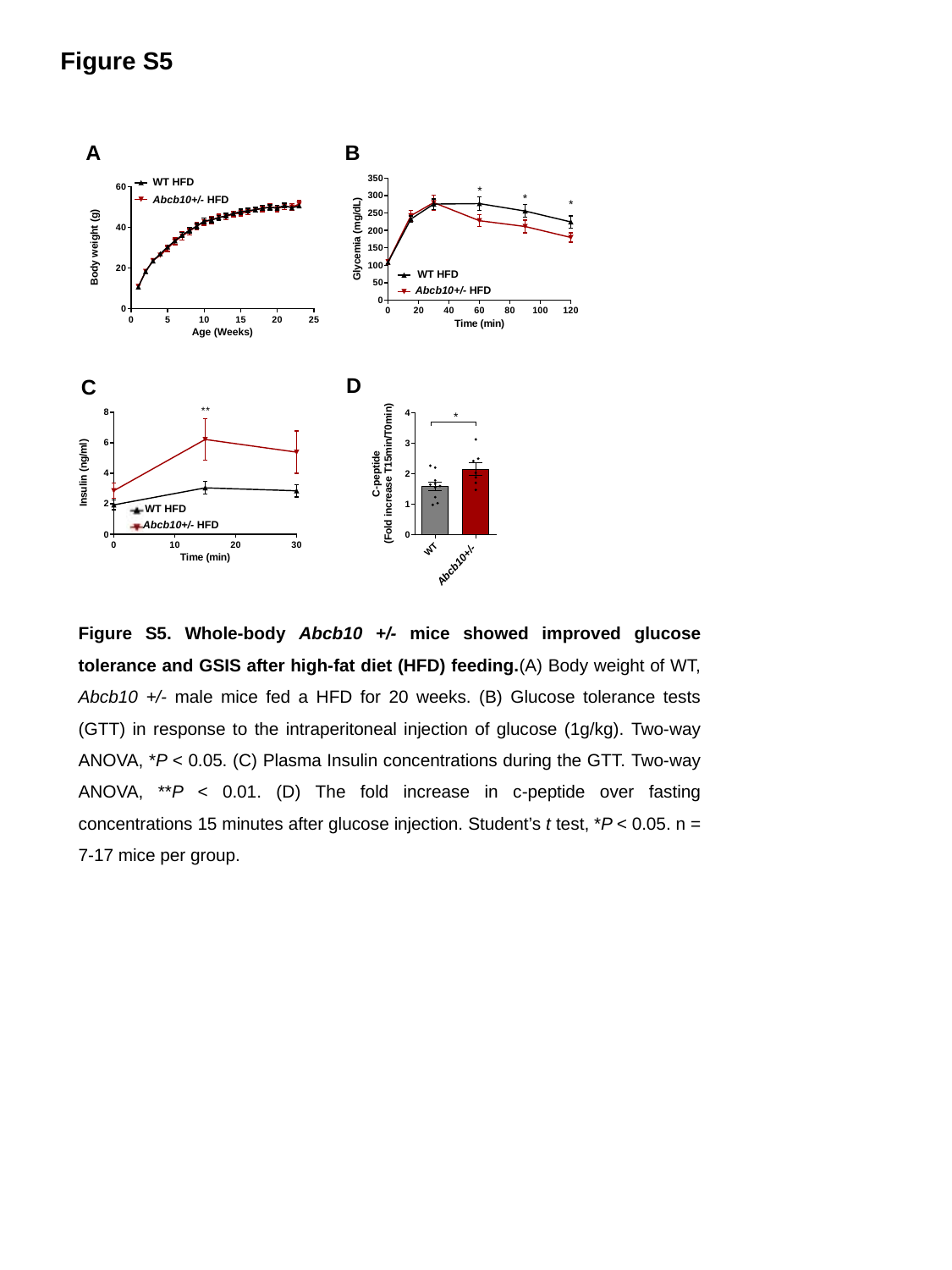

Figure S5
A
B
WT HFD
Abcb10+/- HFD
WT HFD
Abcb10+/- HFD
D
C
WT HFD
Abcb10+/- HFD
Abcb10+/-
Figure S5. Whole-body Abcb10 +/- mice showed improved glucose tolerance and GSIS after high-fat diet (HFD) feeding.(A) Body weight of WT, Abcb10 +/- male mice fed a HFD for 20 weeks. (B) Glucose tolerance tests (GTT) in response to the intraperitoneal injection of glucose (1g/kg). Two-way ANOVA, *P < 0.05. (C) Plasma Insulin concentrations during the GTT. Two-way ANOVA, **P < 0.01. (D) The fold increase in c-peptide over fasting concentrations 15 minutes after glucose injection. Student’s t test, *P < 0.05. n = 7-17 mice per group.

## Slide 6
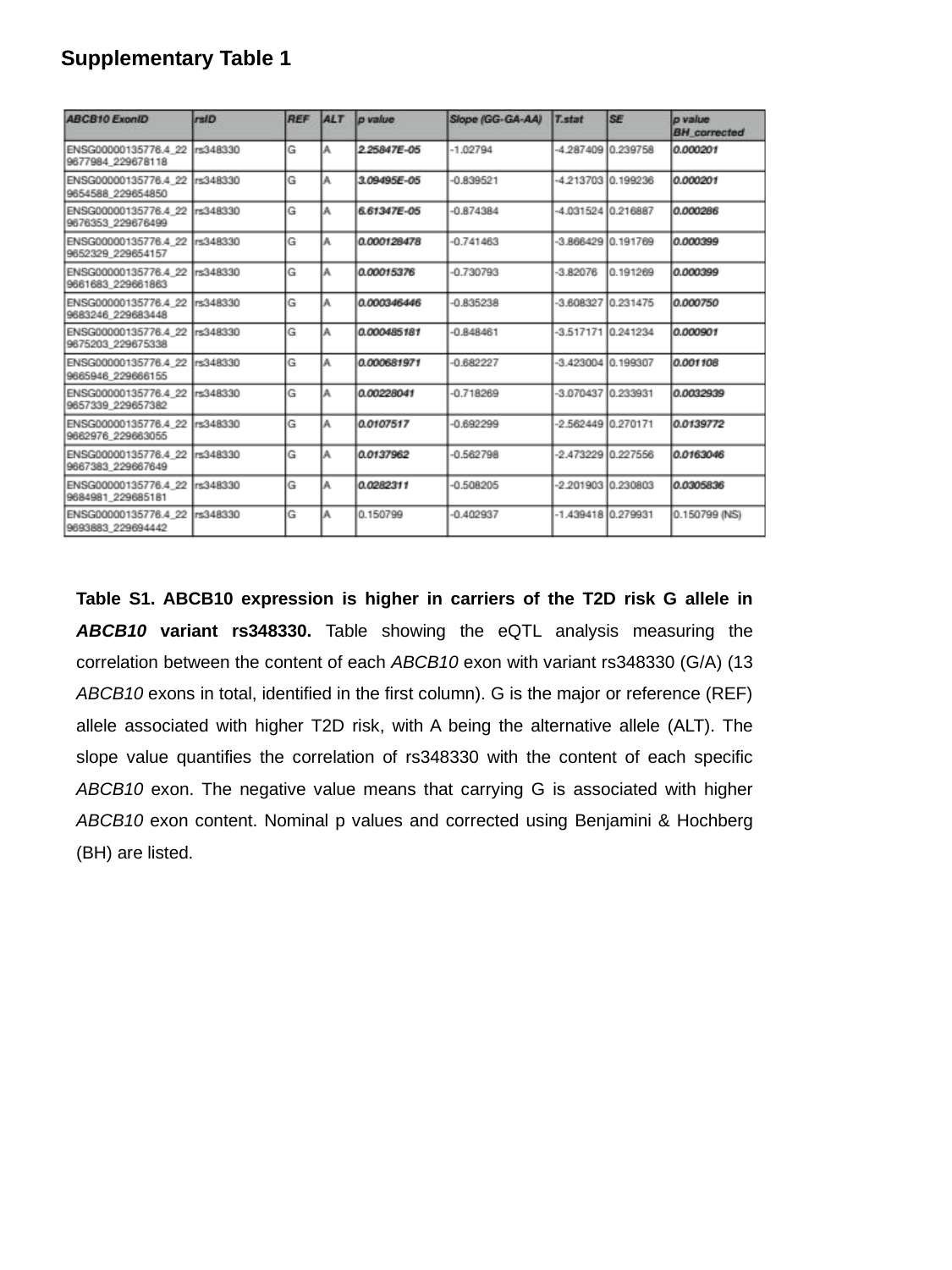

Supplementary Table 1
Table S1. ABCB10 expression is higher in carriers of the T2D risk G allele in ABCB10 variant rs348330. Table showing the eQTL analysis measuring the correlation between the content of each ABCB10 exon with variant rs348330 (G/A) (13 ABCB10 exons in total, identified in the first column). G is the major or reference (REF) allele associated with higher T2D risk, with A being the alternative allele (ALT). The slope value quantifies the correlation of rs348330 with the content of each specific ABCB10 exon. The negative value means that carrying G is associated with higher ABCB10 exon content. Nominal p values and corrected using Benjamini & Hochberg (BH) are listed.
